# Objektive und subjektive Bewertung eines strukturierten Vorgehens zur Beurteilung von Computertomographien der Nasennebenhöhlen

**DOI:** 10.1007/s00106-020-00889-y

**Published:** 2020-05-19

**Authors:** T. Hildenbrand, A. Krahe, M. C. Ketterer, C. Offergeld

**Affiliations:** Klinik für Hals‑, Nasen- und Ohrenheilkunde, Universitätsklinikum Freiburg, Killianstr. 5, 79106 Freiburg, Deutschland

**Keywords:** Endoskopische Nasennebenhöhlenchirurgie, Weiterbildungsassisten*innen, Präoperative CT-Analyse, Anatomische Normvarianten, CLOSE-Kriterien, Endoscopic sinus surgery, Residents in training, Preoperative CT evaluation, CLOSE criteria, Anatomic variants

## Abstract

**Hintergrund:**

Die Voraussetzung zur Vermeidung potenziell schwerwiegender Komplikationen in der Nasennebenhöhlenchirurgie ist eine fundierte Kenntnis der individuellen Anatomie. Für die Nasennebenhöhlen stellt die Computertomographie (CT) die Bildgebung der Wahl dar, um die individuelle Anatomie und das Ausmaß der Erkrankung präoperativ darzustellen.

**Ziel der Arbeit:**

Das Ziel der vorliegenden Arbeit ist die Bewertung des Nutzens einer CT-Checkliste zur Identifikation wichtiger anatomischer Normvarianten in der CT der Nasennebenhöhlen.

**Material und Methoden:**

Jung- und Altassistent*innen der HNO-Klinik wurden gebeten, CTs der Nasennebenhöhlen vor und nach der Einführung der CLOSE-Kriterien auf anatomische Normvarianten zu untersuchen. Der Prozentsatz der korrekt identifizierten Normvarianten wurde berechnet. Zusätzlich wurde der subjektive Nutzen anhand eines Fragebogens beurteilt.

**Ergebnisse:**

Sechs Jung- und 6 Altassistent*innen wurden in die Studie eingeschlossen. Die Rate der korrekt identifizierten Normvarianten verbesserte sich nach der Einführung der CLOSE-Kriterien signifikant von 23,1 auf 50,9 % bzw. von 24 auf 39,8 %. Die subjektive Beurteilung der CLOSE-Checkliste war sehr positiv.

**Schlussfolgerung:**

Ein strukturiertes Vorgehen zur Analyse von CTs der Nasennebenhöhlen kann die Identifikation kritischer anatomischer Strukturen verbessern und wird von Weiterbildungsassistenten als sehr hilfreich bewertet.

## Abstract (en)

**Background:**

Sound knowledge of individual anatomy is essential in sinus surgery to prevent potentially serious complications. For the paranasal sinuses, computed tomography (CT) is the imaging technique of choice to preoperatively analyze individual anatomy and the extent of disease.

**Objective:**

The purpose of this study was to evaluate the usefulness of a CT checklist to identify relevant anatomic variants in CTs of the paranasal sinuses.

**Materials and methods:**

Junior and senior otolaryngology residents were asked to assess sinus CT scans for anatomic variants before and after implementation of the CLOSE mnemonic (cribriform plate, lamina papyracea, Onodi cell, sphenoid sinus pneumatization, and [anterior] ethmoidal artery). The rate of correctly identified variants was calculated. A questionnaire was distributed for subjective evaluation of the usefulness of the checklist.

**Results:**

Six junior and six senior residents were included in the study. The rate of correctly identified anatomic variations significantly improved from 23.1 to 50.9% and 24 to 39.8%, respectively, after implementation of the CLOSE mnemonic. The subjective evaluation of the CLOSE criteria showed very positive results.

**Conclusion:**

The structured approach to sinus CT scans using CT criteria can improve identification of critical anatomic variants in CT scans of the paranasal sinuses and is rated highly positively by residents in training.

## Hintergrund

Die endonasale endoskopische Nasennebenhöhlenchirurgie kann als Goldstandard der chirurgischen Therapie der meisten benignen und malignen Erkrankungen der Nasennebenhöhlen und der Frontobasis angesehen werden, wobei die chronische Rhinosinusitis (CRS) die häufigste Indikation darstellt.

Schwerwiegende Komplikationen wie Schädelbasisverletzungen mit Liquorrhö, intrakranielle Komplikationen, orbitale Komplikationen (z. B. Doppelbilder, Visusminderung) und schwerwiegende Blutungen treten in 0,4–1 % der Fälle auf [4, 5, 9, 15–17]. Die präoperative Computertomographie (CT) der Nasennebenhöhlen soll durch Darstellung der individuellen Anatomie und möglicher Normvarianten helfen, die Komplikationsrate zu reduzieren. So ist auch bei Kindern, unter Berücksichtigung strahlenhygienischer Gesichtspunkte, eine präoperative Bildgebung zu fordern (z. B. Magentresonanztomographie [MRT] bei umschriebenen Eingriffen, bei komplexeren und ausgedehnteren Eingriffen CT).

Checklisten, wie sie in der Luftfahrt- und Automobilindustrie verbreitet sind, werden inzwischen in vielen chirurgischen Fächern eingesetzt, werden von der WHO empfohlen und können durch die Minimierung vermeidbarer Fehler die Sicherheit erhöhen und das Ergebnis verbessern [7, 16]. Verschiedene Checklisten wurden speziell für die Nasennebenhöhlenchirurgie entwickelt, von denen einige die präoperative Analyse von CT-Bildern einschließen. Einige sind sehr detailliert und komplex, und einige beinhalten allgemeine Sicherheitsaspekte [5, 10–15, 18].

Die bewusste Wahrnehmung der individuellen Anatomie und die Erkennung von anatomischen Normvarianten im präoperativen CT können durch diese CT-Checklisten verbessert werden. [5, 12, 18]. Sie können zudem eine sinnvolle Ergänzung für die Ausbildung von Assistenzärzt*innen darstellen [18].

Die Ausbildung in der Nasennebenhöhlenchirurgie erfordert ein detailliertes Verständnis der Anatomie der Nasennebenhöhlen und Schädelbasis. Es gibt nur wenige Studien, die sich mit den Erfordernissen der chirurgischen Ausbildung in der Nasennebenhöhlenchirurgie beschäftigen. Während des Erlernens der Technik wird die Handhabung des Endoskops (insbesondere bei gewinkelten Optiken) und der Instrumente, die räumliche Orientierung mit Übertragung der zweidimensionalen Anatomie aus Lehrbüchern und den CT-Bildern auf den dreidimensionalen Patienten, die Identifikation der bekannten Anatomie im endoskopischen Bild und die Beurteilung der Position des Endoskops und der Instrumente als problematisch angesehen [1, 2].

Checklisten werden in der medizinischen Lehre hauptsächlich im Rahmen von leistungsbasierten Prüfungen, wie OSCE (Objective Structured Clinical Examination), eingesetzt. Hofer et al. führten eine Checkliste für die anatomische Präparation während des Medizinstudiums ein. Sie konnten zeigen, dass sich die Qualität der Präparation und die Ergebnisse während der Testate nach der Einführung verbesserten [9].

Das Kürzel CLOSE zur Identifikation wichtiger anatomischer Strukturen in der CT der Nasennebenhöhlen wurde von Weitzel eingeführt [17]. Es ist plausibel und nachvollziehbar. Es beinhaltet die wichtigsten anatomischen Strukturen, die Gefahrenpunkte während der Operation darstellen.

- „*C*ribriform plate“: Keros-Klassifikation, Asymmetrie, knöcherne Dehiszenz der Schädelbasis
- *L*amina papyracea: Dehiszenz, Prolaps von Orbitainhalt, infraorbitale Zelle, Processus uncinatus mit Kontakt zur Lamina papyracea
- *O*nodi-Zelle (sphenoethmoidale Zelle): vorhanden/nicht vorhanden, Verlauf des N. opticus in Onodi-Zelle
- „*S*phenoid sinus“ (Keilbeinhöhle): Pneumatisation, Dehiszenz des Karotis- und/oder Optikuskanals, Keilbeinhöhlenseptum mit Ansatz am Karotiskanal
- „*E*thmoidal artery“ (A. ethmoidalis anterior): Identifikation des Eintritts in das Siebbein (zipflige Ausziehung an der medialen Wand der Orbita), Verlauf durch das Siebbein (in der Schädelbasis, frei durch das Siebbein verlaufend)

Das Ziel dieser Arbeit war zu prüfen, ob die Einführung einer CT-Checkliste mit Abarbeitung der CLOSE-Kriterien die Erkennung wichtiger anatomischer Normvarianten in der CT der Nasennebenhöhlen verbessert. Das zweite Ziel war die subjektive Bewertung dieser Kriterien durch HNO-Assistenzärzt*innen in der Weiterbildung.

## Material und Methoden

Jung- und Altassistent*innen der Klinik für Hals‑, Nasen- und Ohrenheilkunde wurden gebeten an der Studie teilzunehmen. Sie wurden darauf hingewiesen, dass die Teilnahme an der Studie freiwillig erfolgt und zu jedem Zeitpunkt beendet werden kann. Die Studie erfolgte im Einklang mit nationalem Recht sowie gemäß der Deklaration von Helsinki von 1975 (in der aktuellen, überarbeiteten Fassung) und wurde durch die örtliche Ethikkommission sowie den Personalrat des Universitätsklinikums genehmigt. Die Assistent*innen erhielten eine schriftliche Information über die Studie und wurden zusätzlich mündlich aufgeklärt. Die Teilnehmer erhielten außerdem Informationen hinsichtlich ihrer Rechte durch die Datenschutzgrundverordnung und stimmten der Verarbeitung und Speicherung ihrer Daten im Rahmen der Studie zu. Die Einwilligung in die Studie und in die Verarbeitung personenbezogener Daten war Voraussetzung für den Einschluss in die Studie.

Als Jungassistent*innen wurden Assistenzärzt*innen im 2.–4. Jahr der Weiterbildung, als Altassistent*innen Assistenzärzt*innen im 5. Jahr der Weiterbildung und Fachärzt*innen definiert.

Die Daten wurden pseudonymisiert erhoben und ausgewertet.

Während eines ersten Termins wurden den Assistenzärzt*innen 10 anonymisierte CT-Datensätze in 2 oder 3 Ebenen (axial, koronar und, wenn vorhanden, sagittal) vorgelegt. Diese sollten auf das Vorhandensein anatomischer Normvarianten geprüft werden. Das Ausmaß der Erkrankung sollte nicht beurteilt werden. Insgesamt waren 18 anatomische Normvarianten in den Bildern zu identifizieren. Dies waren 2 Normvarianten der Lamina cribrosa, 6 der Lamina papyracea, 4 sphenoethmoidale Zellen, 2 Conchae bullosae, 2 Anomalien der Keilbeinhöhle und 2 der A. ethmoidalis anterior. Die CT-Datensätze wurden in einem den Assistent*innen aus der täglichen Routine bekannten CT-Viewer dargestellt, in dem die Teilnehmer*innen durch die verschiedenen Ebenen scrollen konnten.

In einer Lehreinheit im Rahmen der abteilungsinternen Fortbildungsveranstaltung wurden die CLOSE-Kriterien eingeführt. Während eines zweiten Termins sollten die Teilnehmer*innen dieselben CT-Datensätze erneut beurteilen, jedoch unter Verwendung der CLOSE-Kriterien als CT-Checkliste. Um das Risiko eines Lerneffekts durch Wiederholung zu reduzieren, wurde den Assistenzärzt*innen nach dem ersten Termin nicht mitgeteilt, ob ihre Angaben richtig waren, und es lagen mindestens 4 Wochen zwischen beiden Terminen. Es gab kein Zeitlimit für die Beurteilung der CT.

Nach dem zweiten Termin füllten die Teilnehmer*innen einen anonymen Evaluationsbogen zum Nutzen der CLOSE-Kriterien aus (modifiziert nach [18]). Die Fragen sind in Tab. 1 zusammengestellt. Die Evaluation erfolgte anhand einer 4‑stufigen Likert-Skala (1 = Stimme ich vollkommen zu, 2 = Stimme ich eingeschränkt zu, 3 = Stimme ich überhaupt nicht zu, 4 = Ich weiß nicht).

**Table 1:** 

Frage 1	Die präoperative Checkliste ist sinnvoll
Frage 2	Die Checkliste stellt sicher, dass ich mir genug Zeit für die Betrachtung der CT-Bilder nehme
Frage 3	Die Checkliste macht mich sicherer im Umgang mit der Anatomie und der Computertomographie der Nasennebenhöhlen
Frage 4	Die Checkliste ist überflüssig, da ich mir die enthaltenen Punkte routinemäßig ansehe
Frage 5	Ich werde die Checkliste auch in Zukunft verwenden
Frage 6	Die präoperative Checkliste ist ein sinnvolles Instrument, um chirurgische Gefahrenpunkte zu identifizieren und die Sicherheit der Operation zu erhöhen

Die korrekt identifizierten anatomischen Normvarianten vor und nach Einführung der CLOSE-Kriterien in Prozent wurden für die gesamte Gruppe und die Untergruppen der Jung- und Altassistent*innen verglichen. 18 Normvarianten wurden als 100 % gewertet.

Die statistische Analyse erfolgte mit IBM SPSS Statistics (IBM SPSS Statistics für Windows, Version 24.0, Fa. IBM Corp. 2015, Armonk, NY, USA).

Der Vergleich der Daten erfolgte mittels Wilcoxon-Test, und der *p*-Wert für statistische Signifikanz wurde auf 0,05 festgelegt. Die Vergleiche wurden mit dem Levene-Test berechnet, um eine homo- vs. heterogene Varianz zu definieren, und mittels T‑Test wurden statistisch signifikante Unterschiede definiert.

Die Grafiken wurden mit Excel (Fa. Microsoft 2010, Redmond, WA, USA) erstellt.

Die Ergebnisse des Evaluationsbogens wurden deskriptiv ausgewertet.

Minimum, Maximum, Mittelwert und Standardabweichung wurden für jede Frage berechnet.

## Ergebnisse

Dreizehn Assistenzärzt*innen wurden initial in die Studie eingeschlossen. Zwölf Assistent*innen nahmen an 2 CT-Auswertungen teil, 6 Jung- und 6 Altassistent*innen. Ein Assistenzarzt konnte keinen zweiten Termin wahrnehmen und wurde von der Auswertung ausgeschlossen. Jeder der Teilnehmer beurteilte 10 CT-Datensätze vor und nach der Einführung der CLOSE-Kriterien. Alle Teilnehmer*innen beantworteten den Evaluationsbogen.

**Figure 1:**
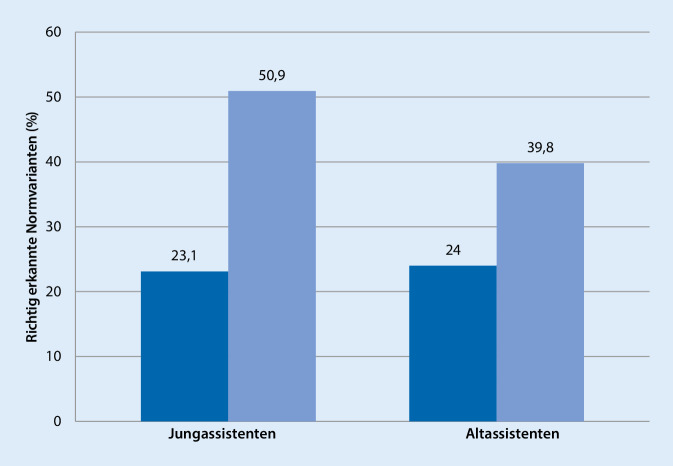

Insgesamt wurden bei der ersten Auswertung 23,6 % der anatomischen Normvarianten richtig erkannt. Jungassistent*innen identifizierten 23,1 %, Altassistent*innen 24 % der anatomischen Normvarianten. Nach der Einführung der CLOSE-Kriterien wurden 45,3 % der Normvarianten von der Gesamtgruppe und 50,9 % von Jung- und 39,8 % von Altassistent*innen richtig erkannt (Abb. 1). Es zeigte sich eine statistisch signifikante Verbesserung der richtig erkannten anatomischen Varianten für die gesamte Gruppe (*p* < 0,0001) und die Untergruppen der Jung- (*p* = 0,003) und Altassistent*innen (*p* = 0,019). Obwohl Jungassistent*innen mithilfe der CLOSE-Kriterien mehr Normvarianten erkannten als Altassistent*innen, zeigte sich kein statistisch signifikanter Unterschied zwischen beiden Gruppen (*p* = 0,263).

Die Ergebnisse der subjektiven Evaluation sind in Tab. 2 zusammengefasst. 91,7 % der Assistentzärzt*innen fanden die CLOSE-Kriterien sinnvoll, und 83,3 % gaben an, dass sie sicherstellen, dass sie sich genug Zeit für die Beurteilung der CT-Bilder nehmen. 75 % der Assistent*innen stimmten vollkommen zu, und 25 % stimmten eingeschränkt zu, dass sie die Kriterien sicherer im Umgang mit der Anatomie und CT-Bildern der Nasennebenhöhlen machen. 83,3 % stimmten nicht zu, dass die CLOSE-Kriterien überflüssig seien. Alle Teilnehmer*innen stimmten vollkommen zu, dass die präoperative Checkliste ein sinnvolles Instrument ist, um chirurgische Gefahrenpunkte zu identifizieren und die Sicherheit der Operation zu erhöhen. Alle Teilnehmer*innen gaben, dass sie die Kriterien weiterhin verwenden werden. Die Tab. 2 zeigt die Ergebnisse der Evaluation mit Mittelwert und Standardabweichung.

**Table 2:** 

	Minimum (Anzahl Assistenten)	Maximum (Anzahl Assistenten)	Mittelwert	SD
*Frage 1*	1 (11)	2 (1)	1,08	±0,29
*Frage 2*	1 (10)	2 (2)	1,17	±0,39
*Frage 3*	1 (9)	2 (3)	1,25	±0,45
*Frage 4*	2 (2)	3 (10)	2,83	±0,39
*Frage 5*	1 (12)	1 (12)	1,00	±0
*Frage 6*	1 (11)	2 (1)	1,08	±0,29

1 = Stimme ich vollkommen zu, 2 = Stimme ich eingeschränkt zu, 3 = Stimme ich überhaupt nicht zu, 4 = Ich weiß nicht

*SD* Standardabweichung

## Diskussion

Unsere Ergebnisse zeigen, dass die Erkennung wichtiger anatomischer Normvarianten in der CT der Nasennebenhöhlen durch die Einführung einer CT-Checkliste zur strukturierten Beurteilung von CT-Bildern verbessert werden kann.

Error et al. konnten ebenfalls eine signifikante Verbesserung der Identifikation wichtiger anatomischer Strukturen in der CT der Nasennebenhöhlen nach Einführung der CLOSE-Kriterien bei Jung- und Altassistent*innen feststellen [5]. In einer einfach verblindeten Studie wurden Assistenzärzt*innen vor und nach der Einführung der CLOSE-Kriterien von einem HNO-Chirurgen vor Beginn einer Operation zum Vorhandensein individueller kritischer anatomischer Strukturen befragt. 57 präoperative Befragungen (28 vor und 29 nach der Einführung) wurden ausgewertet. Mit einer Gesamtzahl von 120 beurteilten CT vor und nach der Einführung der CLOSE-Kriterien bewertet unsere Studie eine wesentlich größere Anzahl. Unsere Studie war nicht verblindet, jedoch hatten die Teilnehmer vor dem ersten Studientermin keine Möglichkeit sich vorzubereiten, da sie ohne vorherige Ankündigung gebeten wurden, an der Studie teilzunehmen. Da wir die Leistung der Teilnehmer*innen mit dem während ihrer täglichen Arbeit und Ausbildung erworbenen Wissen beurteilen wollten, erhielten sie zuvor keine Lehreinheit zur CT-Anatomie der Nasennebenhöhlen. Wir nahmen zudem eine subjektive Evaluation in die Beurteilung der CLOSE-Kriterien mit auf. Die Checkliste wurde von den Assistenzärzt*innen positiv beurteilt. Eine Studie von Yao untersuchte den pädagogischen Wert und die Effektivität einer präoperativen CT-Checkliste aus der Perspektive von HNO-Ärzt*innen in der Weiterbildung [18]. Die Ergebnisse sind mit unseren vergleichbar. Die meisten Teilnehmer*innen stimmten zu, dass die genutzte Checkliste sinnvoll sei, sie sicherer im Umgang mit der CT-Anatomie der Nasennebenhöhlen mache und helfe, kritische anatomische Strukturen zu identifizieren. Die teilnehmenden Ärzt*innen nutzten die Checkliste auch nach Beendigung der Rotation weiter. Unsere Assistenzärzt*innen gaben an, dass sie die Kriterien weiterhin nutzen werden, was wir allerdings noch nicht überprüft haben.

In einer Umfrage an deutschen HNO-Kliniken zeigte sich, dass die meisten Ärzt*innen in der Weiterbildung zwischen dem 2. und 4. Jahr der Weiterbildung mit der Nasennebenhöhlenchirurgie beginnen und dass die meisten an einem Operationskurs zur Nasennebenhöhlenchirurgie teilnehmen, um sich mit der Anatomie und chirurgischen Technik vertraut zu machen [8]. In der Regel beginnen sie mit einfachen Eingriffen, wie einer Uncinektomie und Kieferhöhlenoperation, um im Verlauf zu komplexeren Eingriffen überzugehen. Diese werden in der Regel durch erfahrenere Ärzt*innen, meist Fachärzt*innen, durchgeführt. In unserer Abteilung gibt es bisher keine einführende Lehrveranstaltung in die CT-Anatomie der Nasennebenhöhlen und keine rhinologische Rotation, während der Assistent*innen mehr Erfahrungen in der Beurteilung von CT der Nasennebenhöhlen sammeln könnten. Dies und der begrenzte Zugang zu komplexeren Eingriffen an den Nasennebenhöhlen könnte die geringe Rate an richtig erkannten anatomischen Normvarianten während des ersten Studientermins erklären, obwohl die Ergebnisse mit denen der Studie von Error übereinstimmen, in der Jungassistent*innen 29 % und Altassistent*innen 22 % der anatomischen Varianten vor der Einführung der CLOSE-Kriterien erkannten, bei einer Gesamtrate von 24 % [5].

Nach der Einführung der CLOSE-Kriterien verbesserte sich die Identifikation der Normvarianten signifikant, blieb jedoch sowohl bei Jung- als auch bei Altassistent*innen deutlich unter der von Error et al. (51 und 40 % vs. 94 und 83 %). Eine Erklärung hierfür könnte sein, dass sich die Teilnehmer*innen in der Studie von Error in einer Rhinologie-Rotation befanden und so mehr Kontakt mit CT der Nasennebenhöhlen hatten. Unsere Ergebnisse deuten darauf hin, dass die Erkennung kritischer anatomischer Strukturen durch eine Checkliste verbessert werden kann, dass jedoch auch Übung und der wiederkehrende Kontakt mit und die Beurteilung von Nasennebenhöhlen-CT, z. B. im Rahmen einer regelmäßigen operativen Tätigkeit, notwendig zu sein scheinen.

Nach der Einführung der CLOSE-Kriterien zeigten die Jungassistent*innen bessere Ergebnisse als die Altassistent*innen, auch wenn der Unterschied nicht signifikant war. Eine Erklärung könnte eine größere Motivation und Bereitschaft der Jungassistent*innen sein, Neues zu lernen und auszuprobieren, was durch Gewohnheit und Nachlässigkeit bei Altassistent*innen verhindert werden könnte. Ein Hinweis darauf könnte sein, dass es sich bei den beiden Teilnehmern, die angaben, dass die CLOSE-Kriterien überflüssig seien, um 2 der 6 Altassistent*innen handelte. Eine bessere Annahme und konsequentere Anwendung der Kriterien in der Auswertung könnte das bessere Abschneiden der Jungassistent*innen erklären.

Eine besondere Schwierigkeit im Erlernen der endoskopischen Nasennebenhöhlenchirurgie ist die Übertragung der CT-Anatomie der Nasennebenhöhlen in die dreidimensionale Realität beim Patienten [1, 2]. Aus diesem Grund scheint es sinnvoll, sowohl die Lehre der CT-Anatomie in das Curriculum für Assistenzärzt*innen aufzunehmen als auch eine CT-Checkliste wie das CLOSE-System systematisch präoperativ anzuwenden, da sie dazu beitragen kann, ein strukturiertes Vorgehen zu erlernen.

Es existieren nicht viele Studien, die den Nutzen von Checklisten in der medizinischen Lehre beurteilen. Die meisten Studien bewerten den Einsatz in strukturierten Prüfungen. Hofer konnte zeigen, dass die Einführung einer Checkliste im Präparationskurs zu einem besseren Lernergebnis und einer besseren Präparationsqualität führte [9]. Wir evaluieren derzeit den Nutzen der CLOSE-Kriterien in der studentischen Lehre in einer weiteren Studie.

Radiologische Checklisten können nicht nur eine strukturierte Beurteilung von CT-Bildern ermöglichen, sie können auch sicherstellen, dass nichts übersehen wird. Im Qualitäts- und Risikomanagement existieren verschiedene Theorien und Modelle zu vermeidbaren Fehlern mit potenziell fatalen Auswirkungen. Eine präventive Methode nennt sich Poka Yoke. Sie wurde von Shigeo Shingo entwickelt. Die zugrunde liegende Annahme ist, dass unbeabsichtigte Fehler durch Nachlässigkeit und Vergesslichkeit entstehen. 3 Poka-Yoke-Methoden sollen diese Fehler verhindern: konstruktive Maßnahmen (z. B. spezielle Sicherheitskanülen, die ein Recapping verhindern), Überwachungsmaßnahmen (z. B. Warnsignal, wenn Tür des Medikamentenkühlschranks offengelassen wird) und Selbsttests. Chirurgische Sicherheitschecklisten sind eine Form der Selbsttestung. Eine gut etablierte chirurgische Checkliste ist die WHO-Checkliste. Die meisten Studien konnten zeigen, dass sie die Komplikationsrate senken und das Ergebnis verbessern kann [3, 4, 6, 7, 16].

Abgesehen von Sicherheitsaspekten können Checklisten auch das Vorhandensein und die Funktionsfähigkeit der Ausrüstung sicherstellen und so die Effizienz steigern. Es wurden spezielle Checklisten für die Nasennebenhöhlenchirurgie vorgeschlagen, und es konnte gezeigt werden, dass sie potenzielle Sicherheitslücken aufdecken können [13–15]. Diese Checklisten beinhalten Punkte zum Vorhandensein und zu der Richtigkeit der vorhandenen CT-Bilder und die Diskussion wichtiger individueller anatomischer Normvarianten. Sie bieten jedoch kein strukturiertes Vorgehen zur Identifikation dieser Varianten. Eine CT-Checkliste muss nicht zwingend in der präoperativen Checkliste integriert sein, sie kann jedoch zur Evaluation der CT-Bilder durch den Operateur vor dem Eingriff und für die Ausbildung von Assistenzärzt*innen und Student*innen genutzt werden.

Die vorliegende Studie war nicht dazu ausgelegt zu beweisen, dass eine Verbesserung der Erkennung kritischer anatomischer Strukturen zu einer Senkung der Komplikationsrate führt. Es scheint allerdings logisch, dass die präoperative Identifikation potenzieller chirurgischer Fallstricke das Auftreten potenzieller Komplikationen verhindern könnte.

Eine Limitation dieser Studie ist, dass wir nicht ausschließen können, dass durch die Wiederholung ein gewisser Lerneffekt zwischen dem ersten und zweiten Studientermin für die Verbesserung der Ergebnisse eine Rolle gespielt hat. Da wir aber eine hochsignifikante Verbesserung der Gesamtgruppe und der Jungassistent*innen sehen, sind wir zuversichtlich, dass diese Verbesserung nicht nur auf eine Wiederholung, sondern auf das strukturierte Vorgehen mithilfe der CLOSE-Kriterien zurückzuführen ist.

## Fazit für die Praxis

- Unsere Ergebnisse zeigen, dass die Identifikation anatomischer Normvarianten in der CT der Nasennebenhöhlen durch eine strukturierte Analyse wichtiger anatomischer Strukturen anhand einer CT-Checkliste verbessert werden kann.
- Dieses Vorgehen wird sowohl von Jung- als auch von Altassistent*innen positiv beurteilt.
- Die systematische Anwendung einer präoperativen CT-Checkliste wie z. B. das CLOSE -System ist zu empfehlen.
